# Supplementary material for: Piscine Orthoreovirus (PRV)-3, but Not PRV-2, Cross-Protects against PRV-1 and Heart and Skeletal Muscle Inflammation in Atlantic Salmon
Source: Vaccines (Basel). 2021 Mar 6;9(3):230. doi: 10.3390/vaccines9030230 (PMC8001985; doi:10.3390/vaccines9030230)
Supplement: Supplementary file 1 [file vaccines-09-00230-s001.zip › vaccines-1105449 supplementary for proof/supplementary/S1 PRV ViVaAct Supplementary table S1.docx]

Table S1. Primer and probe sequences (5’-3’) for PRV subtypes and immune genes

| PRV subtypes | Primer/Probe | Sequence (5’-3’) | Concentration |
| --- | --- | --- | --- |
| PRV-1 (S1) [38] | Fwd | TGCGTCCTGCGTATGGCACC | 400nM |
|  | Rev | GGCTGGCATGCCCGAATAGCA |  |
|  | Probe (FAM) | ATCACAACGCCTACCT | 300nM |
| PRV-2 (S1) [22] | Fwd | GTCGTTTGATGGAGATGATGGA | 500nM |
|  | Rev | TACATGACGATGGGCAAAGG |  |
| PRV-3 (S1)[24] | Fwd | TCGTGGTTCCAATGACAG | 500nM |
|  | Rev | CCAACCACTAAAACCGAG |  |
|  | Probe (FAM) | ACGCCTTAGAGACAACATGCGAAG | 300nM |
| Immune genes | **Primer/Probe** | **Sequence (5’-3’)** | **Concentration** |
| CD8α | Fwd | GTCTACAGCTGTGCATCAATCAA | 500nM |
|  | Rev | GGCTGTGGTCATTGGTGTAGT |  |
| IFNγ | Fwd | AAGGGCTGTGATGTGTTTCTG | 500nM |
|  | Rev | TGTACTGAGCGGCATTACTCC |  |
| Granzyme A | Fwd | TAAAGGTCGCATCCCTCATC | 500nM |
|  | Rev | TCCAGACACTGAGCAGTTGG |  |
| Viperin | Fwd | AGCAATGGCAGCATGATCAG | 500nM |
|  | Rev | TGGTTGGTGTCCTCGTCAAAG |  |
| Mx1 | Fwd | GGTGATAGGGGACCAGAGT | 500nM |
|  | Rev | CTCCTCACGGTCTTGGTAGC |  |
| ISG15 | Fwd | ATATCTACTGAACATATATCTATCATGGAAACTC | 500nM |
|  | Rev | CCTCTGCTTTGTTGTGGCCACTT |  |
| EF1ab | Fwd | TGCCCCTCCAGGATGTCTAC | 500nM |
|  | Rev | TCACCAGGCATAGCCGATTC |  |
