# Supplementary figures and images for: Piscine Orthoreovirus (PRV)-3, but Not PRV-2, Cross-Protects against PRV-1 and Heart and Skeletal Muscle Inflammation in Atlantic Salmon
Source: Vaccines (Basel). 2021 Mar 6;9(3):230. doi: 10.3390/vaccines9030230 (PMC8001985; doi:10.3390/vaccines9030230)

Figure S1. Infection level of PRV genetic variants, control of cross-infection.


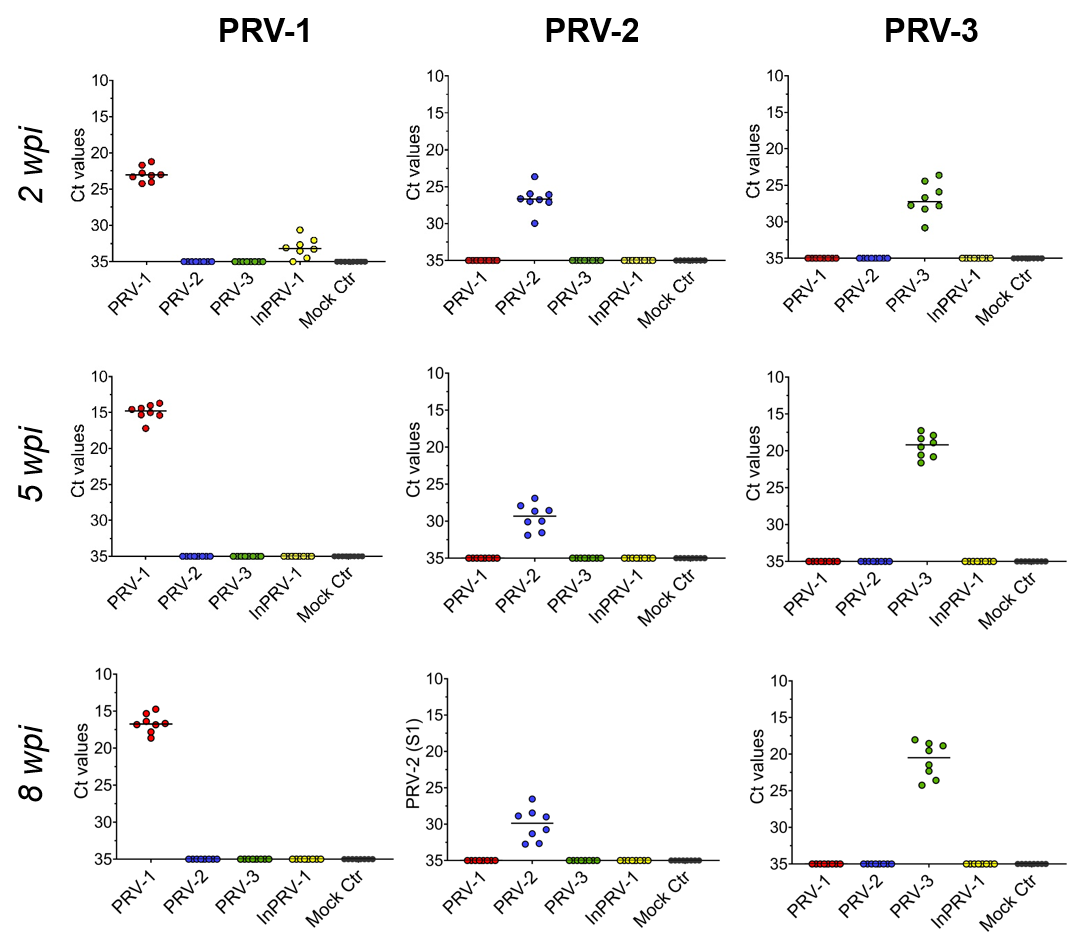

Supplement: Supplementary file 1 [file vaccines-09-00230-s001.zip › vaccines-1105449 supplementary for proof/supplementary/S3 PRV ViVaAct Supplementary Figure S1.docx]
